# Study on dielectric properties of high organic sulfur coking coal and modeling sulfur compounds

**DOI:** 10.1371/journal.pone.0208125

**Published:** 2019-01-03

**Authors:** Chuanchuan Cai, Tao Ge, Mingxu Zhang

**Affiliations:** Department of Material Science and Engineering, Anhui University of Science and Technology, Huainan, PR China; University of New England, AUSTRALIA

## Abstract

Coking coal is geologically scarce resource and most of them cannot be directly used in steel making due to their higher sulfur content. One desulfurization method that has great potential for massive application is microwave desulfurization, which removes the relatively stubborn organic sulfur under mild conditions. The dielectric properties of coals determine the efficiency of the microwave energy absorption. The key to describing the mechanism of microwave desulfurization and further improvement of the desulfurization efficiency is the dielectric response of organic sulfur compounds in coal to microwave. This study focuses on existing formand microwave response of organic sulfur components of three typical coking coal in China. Resultsshowed that the major organic sulfur in selected coals is thiophene which has a stable structure and is the most difficult to be removed. Several dielectric peaks (dielectric loss)andsignificant differencesofeach selected coal samples are observed. The microwave absorption peaks of the model sulfur compounds are identified to be within 9-11GHz. The real parts of the relative dielectric constants (hereinafter referred to as *ε*′) shows a decreasing trend as: diphenyl sulfoxide > diphenyl sulfone > diphenyl sulfide > dibenzothiophene > Octadecane thiol. Response to microwaveare observed to be distinctively different between sulfur-containing and sulfur-free model compounds. The dielectric polarization of mixture (coal mixed with model sulfur compounds) is greater than pure coal. Meanwhile the higher the sulfur content of the coal, the greater the *ε*′ is. Sulfur componentsin coal can significantly influence its polarization.

## Introduction

Coking coal is a crucial raw material for steel industry. High quality coking coal (low sulfur contains) is scarce all around the world, therefore more and more high sulfur coking coal are used instead [1]. Sulfur residues in coke from coking coals can potentially affect the productivity and the quality of steel products [2]. Sulfur emission is also one of the main pollutions to environmental. Therefore, desulfurization technologies of coking coals will bring in significant economic and social impacts. Coal desulfurization by microwave irradiation is a relatively new sulfur removal method in which desulfurization is achieved by harnessing the differences in microwave response among the various sulfur components in coal [3–5]. In recent years, microwave desulfurization research has achieved certain development. The key point of this technology is to know the dielectric properties of the coal and other sulfur-containing components.

Research on the dielectric properties of coal and sulphur-containing constituents is the basic problem of the practice of coal desulfurization with microwave [6]. The dielectric constant determines the behavior of the material under the microwave radiation. The dielectric response of a substance is commonly presented as complex permittivity (ε*), which can be given by:

$${Ɛ}^{*}={Ɛ}^{\prime }-j{Ɛ}^{\prime \prime }$$ (1)

where, *ε*′ is the real part, generally known as dielectric constant (a measure of the ability of the dielectrics to store electrical energy), *ε*″ is the imaginary part, also called the dielectric loss factor, represents the ability of the material to absorb or dissipate the electric energy, j is the imaginary unit.

Studies of dielectric properties of coal have examined the degree of deterioration of coal, the content of moisture and ash, the frequency and temperature during the test and organic sulfur types. The coal with a lower degree metamorphism has a higher dielectric constant. The increase of the coalification degree, the dielectric constant of coal decreases [7]. Coal samples with high ash content have greater dielectric constant. The dielectric constant of the coal sample increases as the test frequency increases [8]. Fe^2+^ and troilite FeSin coal pyrite were oxidized to Fe_2_O_3_ and Fe^3+^ after microwave treatment, indicates a high possibility for coal desulfurization through method enhanced with microwave energy [9]. The dielectric loss of organic sulfur compounds has a significantly higher rate at a frequency of 915 MHz than of 2450 MHz [10]. Moisture and mineral are likely to have increased the dielectric constant of the bulk coal, and low-rank coal’s dielectric constant is higher due to its higher moisture concentration [11]. Relative dielectric constants of two lignite coal samplesobviously increased at elevated temperatures under high-temperature pyrolysis at 800°C heated by 2450 MHz microwave irradiation [12]. The presence of organic sulfur during coal desulfurization creates complexities which make it difficult to accurately determine dielectric properties. Sulfur model compounds have been used to investigate desulfurization. In most recent studies, model sulfur compounds have higher dielectric constants than compounds of similar structure but sulfur free. This study confirmed the feasibility of desulfurization with microwave radiation which, at a proper frequency, can activate sulfur bonds to and cause decomposition of their model compounds [13]. Coking coal is a dielectric with good wave-absorbing properties; 0.3~2.5GHz and 16.5~25GHz are the significant microwave response intervals for coking coal; at the frequency points of 915MHz and 22125MHz, the coking coal has stronger absorbing and transforming capacity towards the microwave [14].

Research into this either studied dielectric properties of coal or sulfur components individually. Few studies investigated dielectric parameters of coking coal and modeling sulfur compounds at the microwave frequency band. To fully understand the mechanisms of coking coal desulfurization by microwave irradiation, it is important to investigate the dielectric properties of the various sulfur components in coking coal. This study will discuss primarily the dielectric properties of high-sulfur coking coal using a microwave source of expanded frequency band. The dielectric properties of organic sulfur compounds and mixtures of these compounds with a low-sulfur coal also been measured. Results will specifically provide a guidance towards the organic sulfur removal by microwave radiation, which is most difficult part of desulfurization.

## Materials and methods

### Coal samples

High-sulfur coking coal samples from Xin Yu, Xin Yang and Xin Liu coal minesinShan xi province have been selected and named as coal A, coal B and coal C, respectively. Coal D from Wang Feng Gang coal preparation plant with 0.1% total sulfur was also selected as low sulfur contain coal. Proximate and elemental analysis, as well as sulfur speciation in selected coals, are carried out and shown in Tables 1–3.

**Table 1:** Proximate analysis of coal samples.

Coal Sample	M_ad_ (%)	A_d_ (%)	V_d_(%)	FC_d_(%)
**A**	1.03	27.81	18.02	54.17
**B**	2.10	29.60	31.54	38.86
**C**	1.20	11.10	25.87	63.03
**D**	0.70	10.95	36.22	52.83

**Table 2:** Elemental analysis of coal samples.

Coal Sample	C_daf_	H_daf_	O_daf_	N_daf_	S_daf_
**A**	84.21	4.45	6.11	1.52	3.71
**B**	85.75	3.31	6.05	0.96	4.11
**C**	85.16	4.53	7.20	1.32	1.79
**D**	84.81	5.71	7.92	1.45	0.11

**Table 3:** Sulfur forms in coal samples.

Coal Sample	S_sd_ (%)	S_p,d_ (%)	S_o,d_(%)	S_t,d_(%)
**A**	0.18	0.76	1.74	2.68
**B**	0.24	0.69	1.97	2.90
**C**	0.10	0.01	1.49	1.60
**D**	0.01	0.03	0.06	0.10

Notes: S_t,d_- total sulfur S_s,d_- sulfate sulfur S_p,d_- pyrite sulfur S_o,d_- organic sulfur

The total sulfur content of both Coal A and B are higher, and Coal C has slightly lower sulfur content. Sulfur types are similar in three high sulfur coal samples. The main sulfur form is organic sulfur, followed by pyrite sulfur and sulfate sulfur content is the lowest.

**Fig 1:**
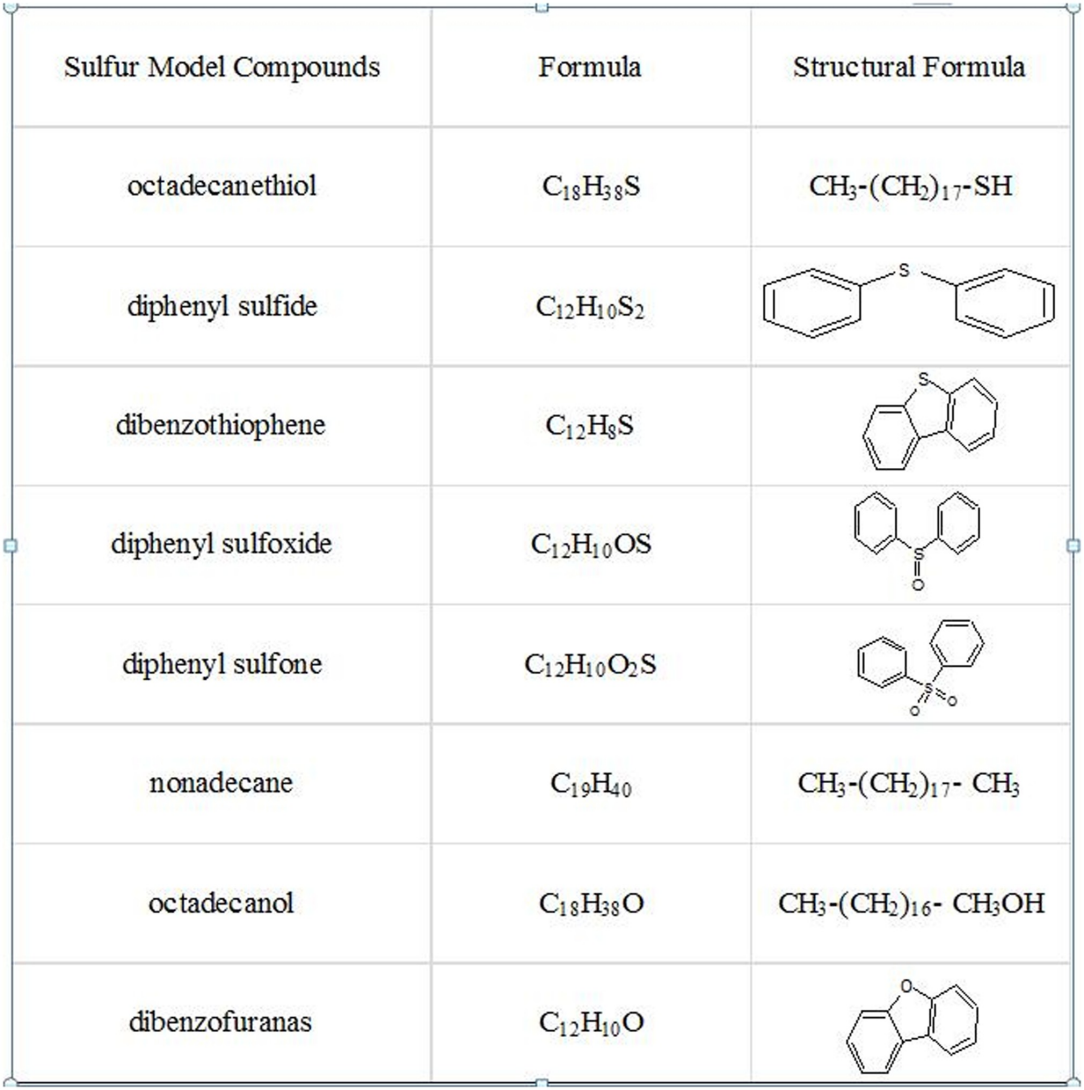
Properties of model compounds.

Five model organic sulfur compounds, previously found in coals, are selected including octadecane thiol, diphenyl sulfide, dibenzothiophene, diphenyl sulfoxide, diphenyl sulfone. The results of studies on the differences in dielectric properties of sulfur-containing model compounds and structurally similar sulfur-free model compounds are of great significance for understanding the role of sulfur-containing components under microwave conditions [13]. For comparison, Nonadecane, octadecanoyl, dibenzofurans are selected due to their similar structure to the model compounds above. Properties of all model compounds are shown in Fig 1. All reagents of analytical grade are purchased from Aladdin Reagent.

### Test methods

#### X-ray photoelectron spectroscopy (XPS)

Qualitative, quantitative, and semi-quantitative chemical analyses are used to evaluate the surface elements of solid samples using XPS. Each coal samples are crushed and ground, then sieved with 200 mesh sieves for the XPS tests. ESCALAB 250Xi X-ray Photoelectron spectrometer is used for XPS test. Binding energy in accordance with the abscissa, the ordinate physiological parameters for Electronic counting curve for result analysis.

#### Dielectric property tests

Methods and samples used for dielectric property test are as follows:

Group 1: Coal samples, including Coal A, Coal B, and Coal C;
Group 2: Sulfur model compounds.
Group 3: Sulfur model compounds mixed with low-sulfur coal D. Coal sample D was grinded into 0.2 mm power and mixed with 5 ml octadecane thiol solution in alcohol. After 15 min ultrasonic oscillation, the solution is dried for one hour dry at constant temperature (40°C).

According to the test requirements of the test method used, all the three groups sample above should be mixed with paraffin wax in the ratio of 1:1, then heated to 70°C in water bath then the samples are pressed into 2 mm thick rings with an outside diameter of 7mm and inside diameter of 3.04 mm for test. Transmissions reflection method is used in this test due to its high accuracy in a broadband. The tests are carried out under the room temperature using Agilent E8363A vector network analyzer. The frequency range is set between 2 to 18 GHz. Real parts (*ε*′) and imaginary parts (*ε*″) of the relative dielectric constants of samples can be obtained by the test and the dielectric loss tangent (tan *σ*) can be calculated.

## Results and discussion

### Identifications of sulfur species in coal using XPS

XPS analysis can provide the identification of specific binding structures of sulfurs in coals and are especially useful in understanding the organic sulfur species. The binding energy of coal samples are obtained by XPS, and PEAK 4.1 software is used for fitting and identifying the involved binding energy peaks of the raw XPS data.

**Fig 2:**
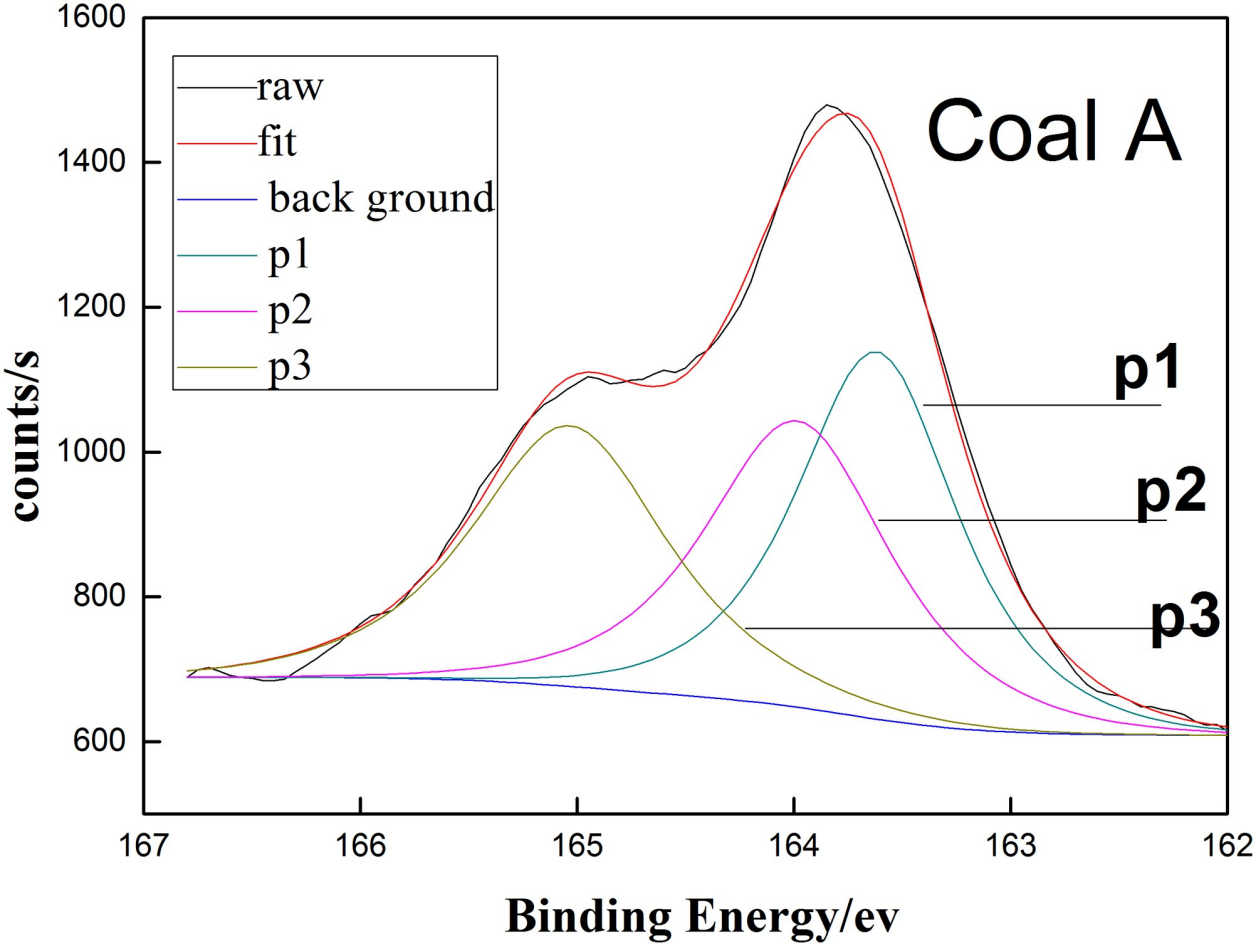
XPS fitting charts Coal A.

Fig 2 present the XPS spectrum of coal A. three split peaks are obtained after the fitting analysis. These three bonding energy peaksare found in coal A, mercaptan(p1), thiophene(p2), and sulfones(p3) [15]. Their specific parameters XPS spectra information of three coal samples are shown in the Table 4.

**Table 4:** XPS parameters of coal samples.

Coal sample	Peak	Position	Area	Percentage/%
**A**	P1	163.61	550.08	37.08
	P2	164.10	477.91	32.21
	P3	165.03	455.30	30.71
**B**	P1	162.01	30.80	2.88
	P2	164.20	716.91	68.78
	P3	165.30	295.30	28.34
**C**	P1	162.01	100.08	10.91
	P2	164.10	521.91	56.89
	P3	165.31	295.30	32.20

Three major organic sulfur species are found in the three coal samples tested, which are mercaptan, thiopheneandsulfone. There is a significant difference in the content of three types of organic sulfur. Contents of thiophene in three selected coals followed a decreasing trend as Coal B (68.78%), Coal C (56.89%) and Coal A (32.21%). And while, contents of mercaptan sulfur ethers followed a decreasing trend as in Coal A (37.08%), Coal C (10.91%) and Coal B (2.88%). As for contents of sulfones, all three coals seemed to be in similar amount at about 30%.

### Dielectric properties analysis

#### Dielectric properties of raw coal samples

**Fig 3:**
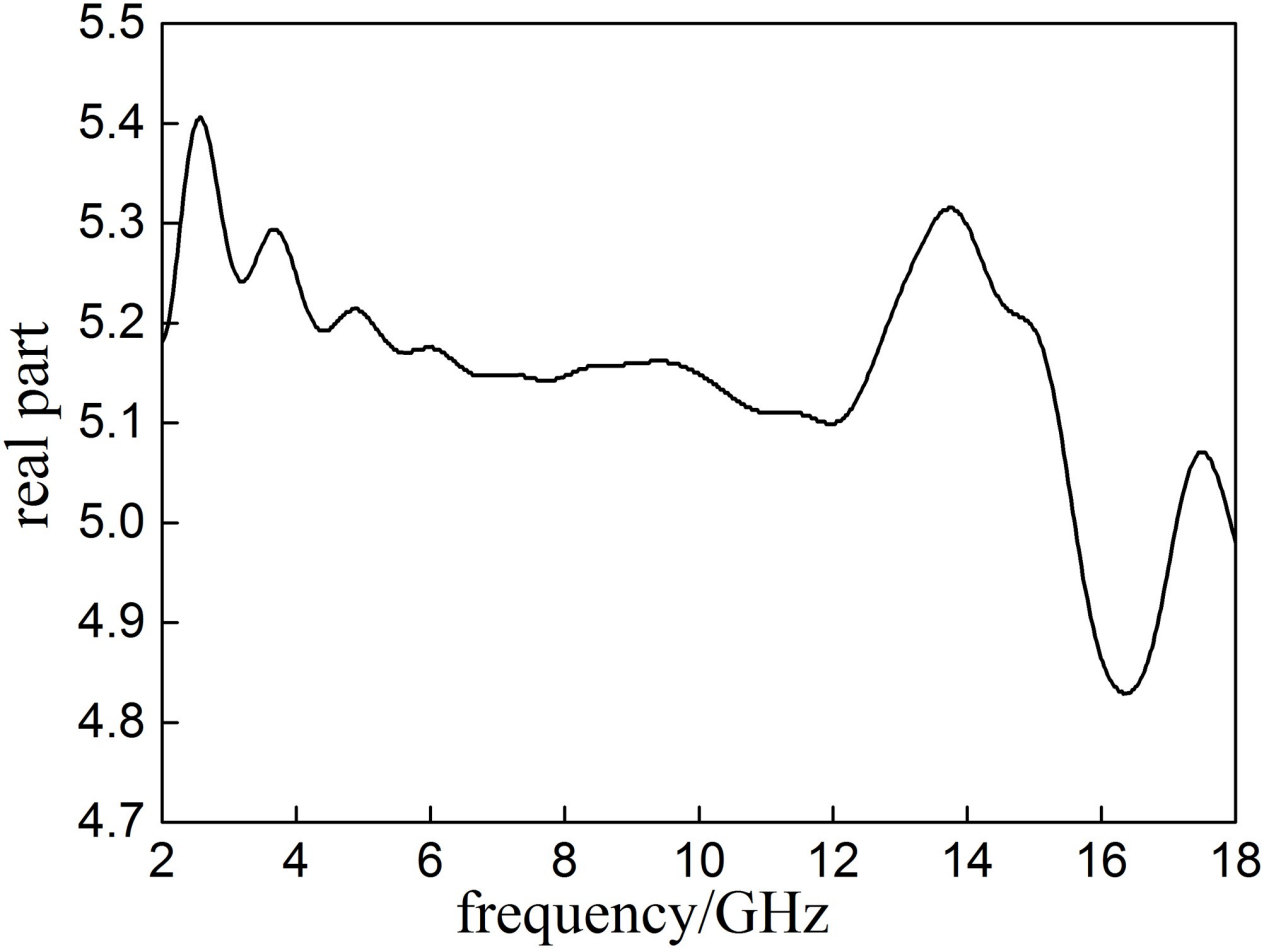
The real partof the complex permeability (*ε*′) of Coal A.

**Fig 4:**
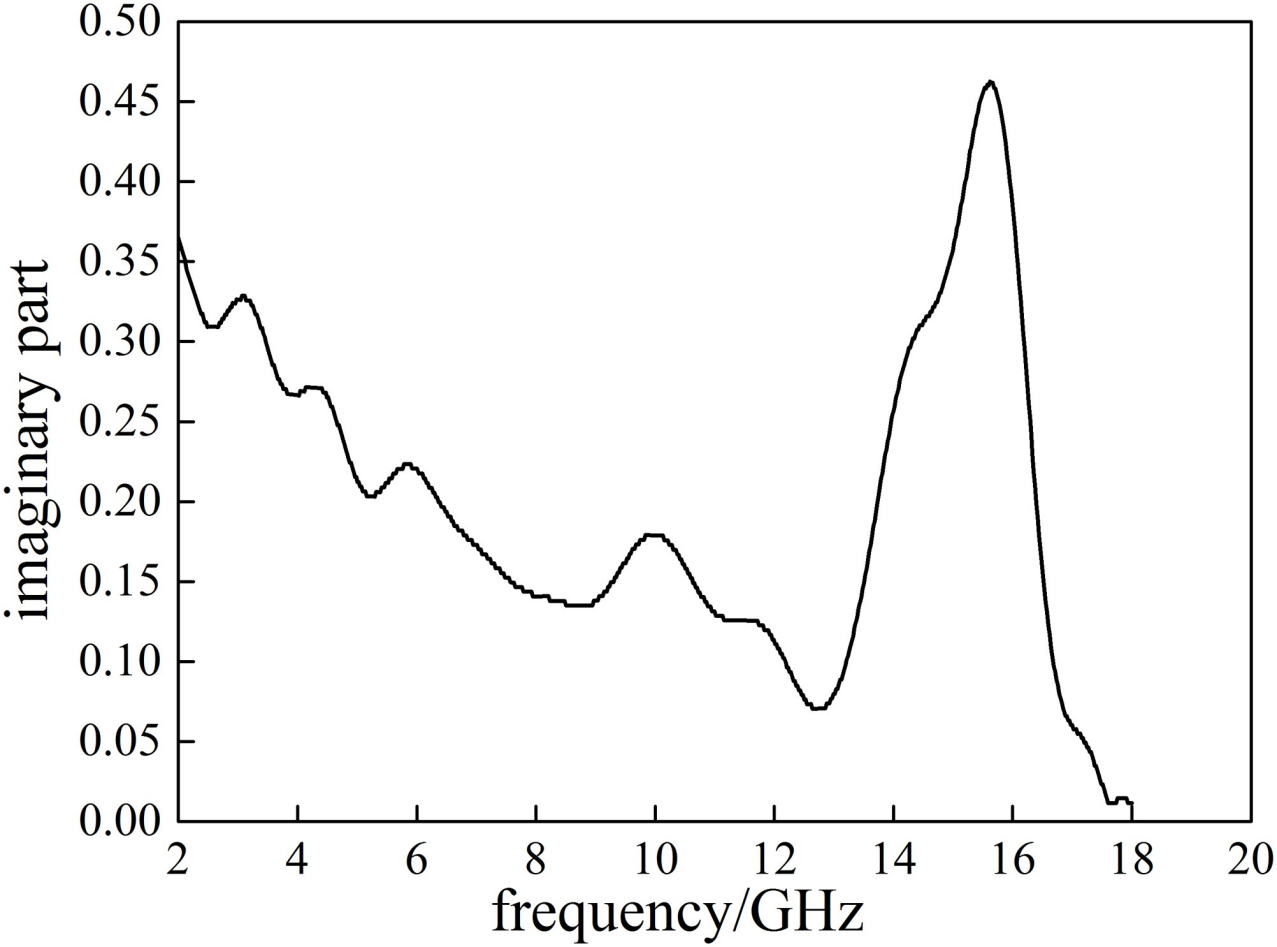
The imaginary part of the complex permeability(*ε*″) of Coal A.

**Fig 5:**
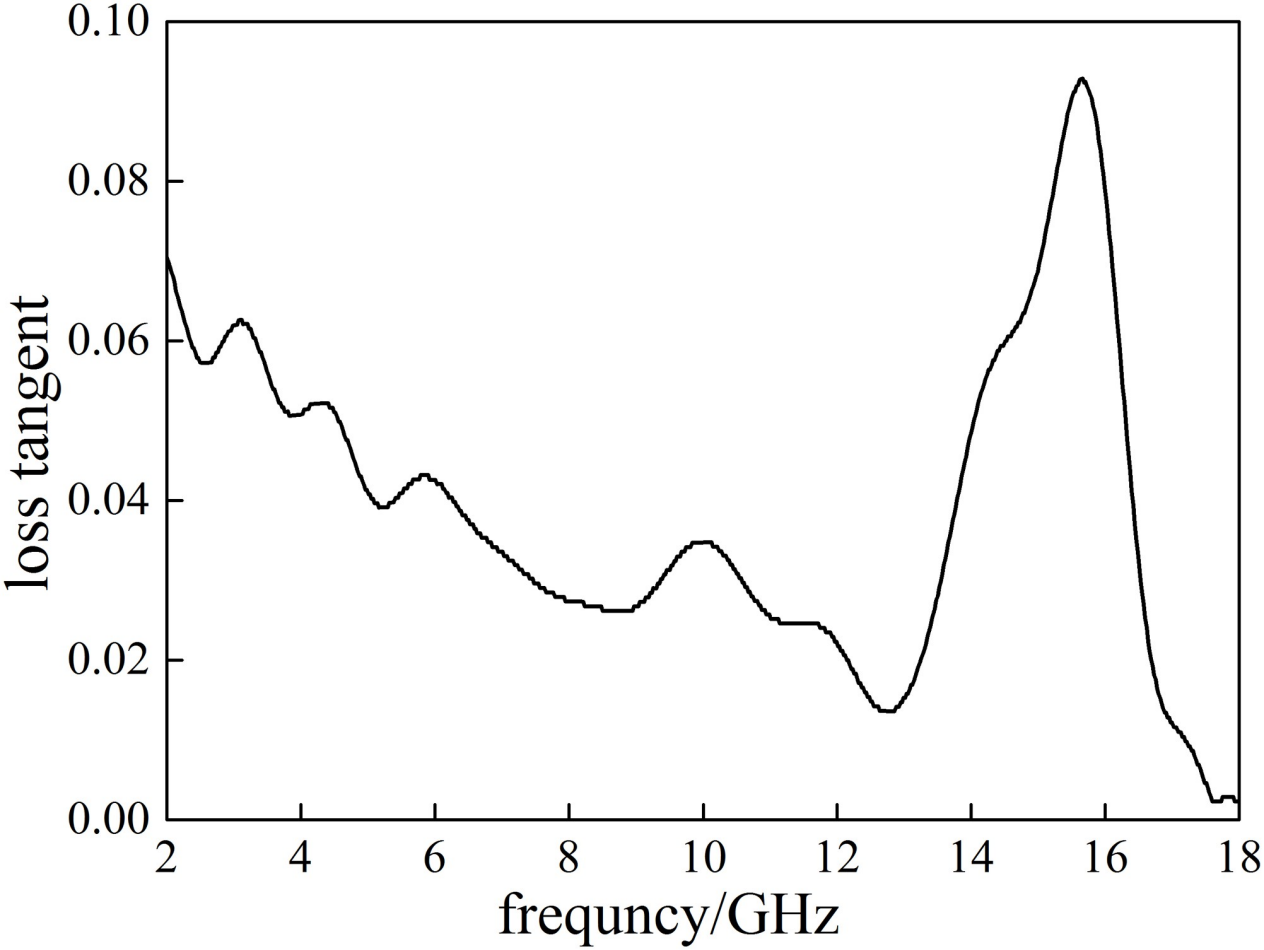
The magnetic loss tangent(tan *α*)plotted of Coal A.

The *ε*′ curve of Coal A are presented in Figs 3–5. Peaks appear in a range between 2–18 GHz, suggesting thatCoal A only responds to the external energy field at specific frequencies. Maximum peak of *ε*′ is at 2.581 GHz, where Coal A has the greatest polarization and greatest response to microwave irradiation.

The imaginary parts of relative dielectric constant (*ε*″) of Coal A decreases first and then increases with the increase of frequency. The highest peak value of *ε*″ is 0.462at 15.619 GHz, where the dielectric loss and the microwaves energy absorption by Coal Ais the greatest. Because the tan *α* of the relative dielectric constants (dielectric loss) of Coal A is greater than 0.01, it can be treated as a dissipative medium [16]. The highest peak of tan *α* appears at 15.664 GHz with a value of 0.093, suggesting the greatest capability of microwave absorption and its ability to convert microwave energy into heat of Coal A at this frequency. The dielectric properties of Coal A, Coal B and Coal C in the same range of frequency (2–18 GHz) are summarized in the Table 5.

**Table 5:** The highest peak information of coal samples.

	*ε*′		*ε*″		tan *α*	
Sample	Peak position(GHz)	Value	Peak position(GHz)	Value	Peak position(GHz)	Value
**Coal A**	2.581	5.405	15.619	0.462	15.664	0.093
**Coal B**	14.68	2.452	16.948	1.044	17.015	0.575
**Coal C**	17.46	2.053	17.151	1.333	17.190	0.580

Table 5 indicates that the maximum value of *ε*′ appears at 14.68GHz and 17.46 GHz for Coal

B and Coal C respectively, where the samples achieve the greatest polarization and response to microwave energy. While the maximum value of *ε*″ of Coal B and Coal C are 1.044 and 1.333, appear at 16.948GHz and 17.151GHz respectively, where the dielectric loss and microwaves energy absorption are the greatest. The highest peaks of tan *α* show 0.575 at 17.015 GHz for Coal B and 0.58 at 17.19 GHz for Coal C. Therefore, both Coal B and Coal C are dissipative mediums.

**Fig 6:**
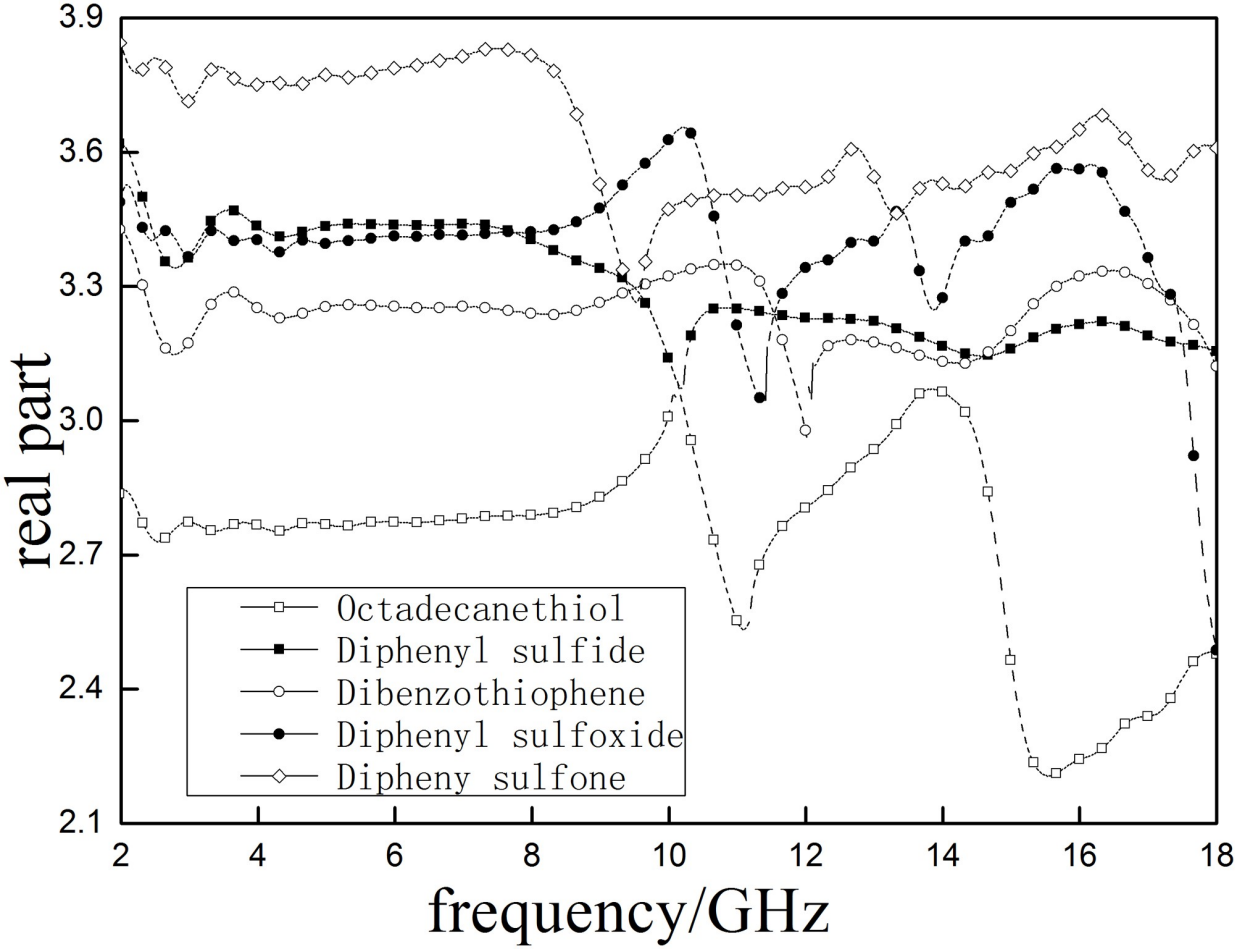
The real part (*ε*′)of the complex permeability of sulfur- containing model compounds.

**Fig 7:**
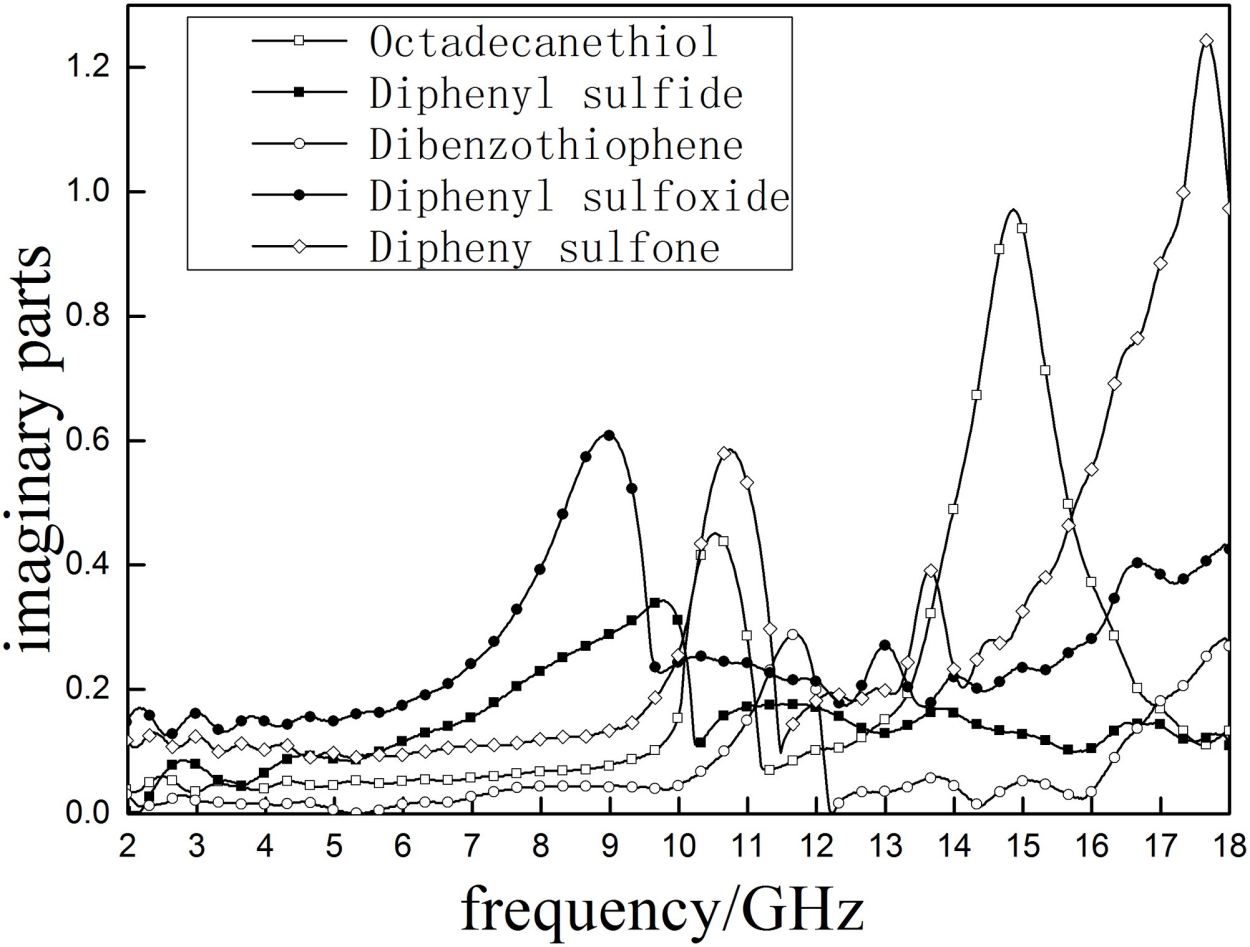
The imaginary part of the complex permeability (*ε*″) of sulfur- containing model compounds.

According to Figs 6 and 7, It can be included that the *ε*′ of five major sulfur-containing model compounds decrease when the frequency increases. Overall, a decreasing trend of sulfur model compounds is observed: diphenyl sulfone > diphenyl sulfoxide > diphenyl sulfide > dibenzothiophene > octadecane thiol. This sequence indicates that *ε*′ will increase when the diphenyl sulfide is oxidized into diphenyl sulfoxide and diphenyl sulfone. The maximum *ε*″ of diphenyl sulfone, Diphenyl sulfoxide and Diphenyl sulfide are 1.24, 0.61 and 0.34 respectively. Results suggest that oxidation treatment will be beneficial to improving the material’s response to microwave energy and increase microwave heating efficiency at certain frequency, which is consistent with Tao’s research [13]. The *ε*′ curve of octadecanethiol has two obvious peaks (10.524 GHz and 14.863GHz) and dibenzothiophene shows three obvious peaks(11.681, 13.75 and 15.063 GHz)within the test range, meaning that they are subjected to relatively large polarization at these peaks. Most of the tested compounds have obvious response in the range 9-13GHz, suggesting that sulfur compound can be easily heated atthis frequency range in the microwave filed.

**Fig 8:**
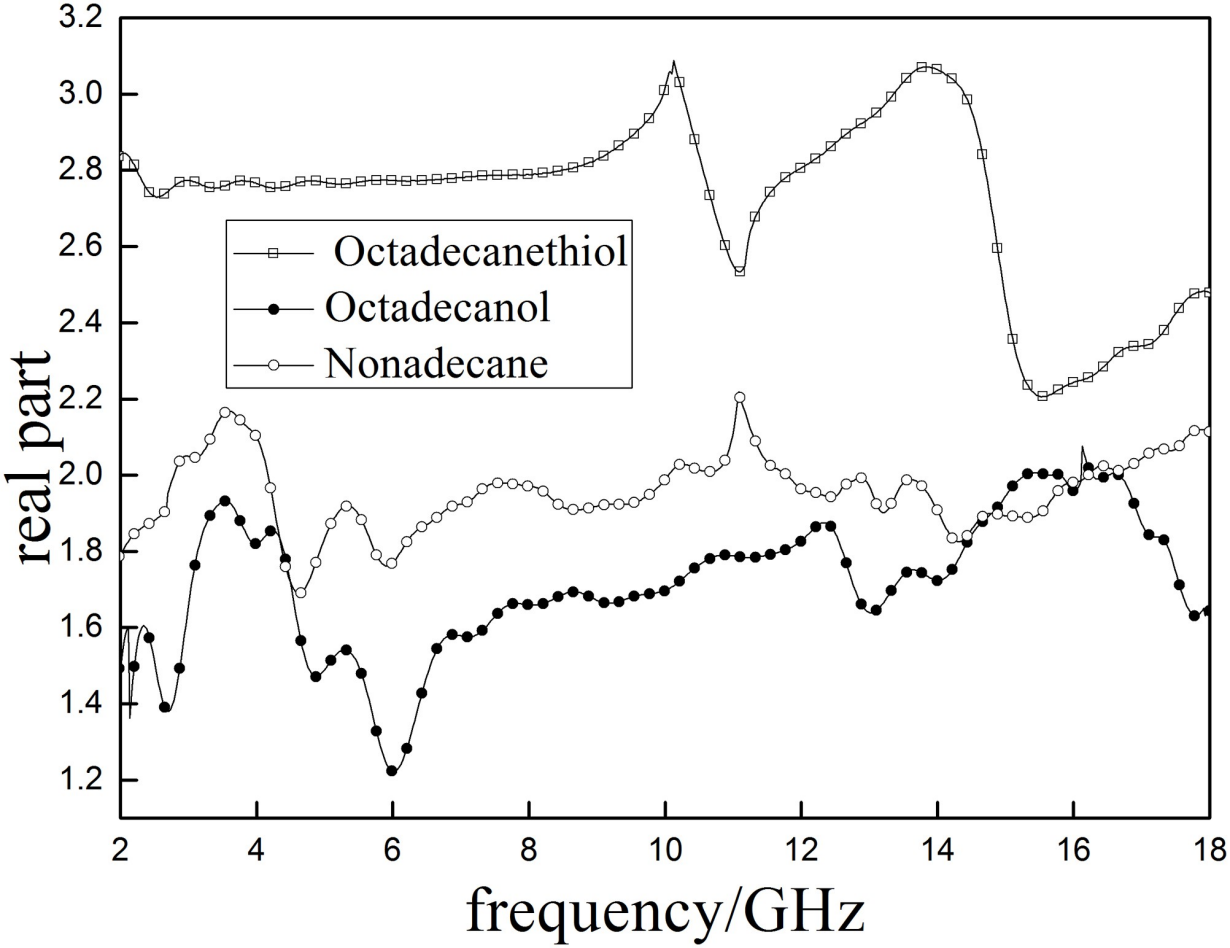
The real part (*ε*′)of the complex permeability of aliphatic model sulfur compounds.

**Fig 9:**
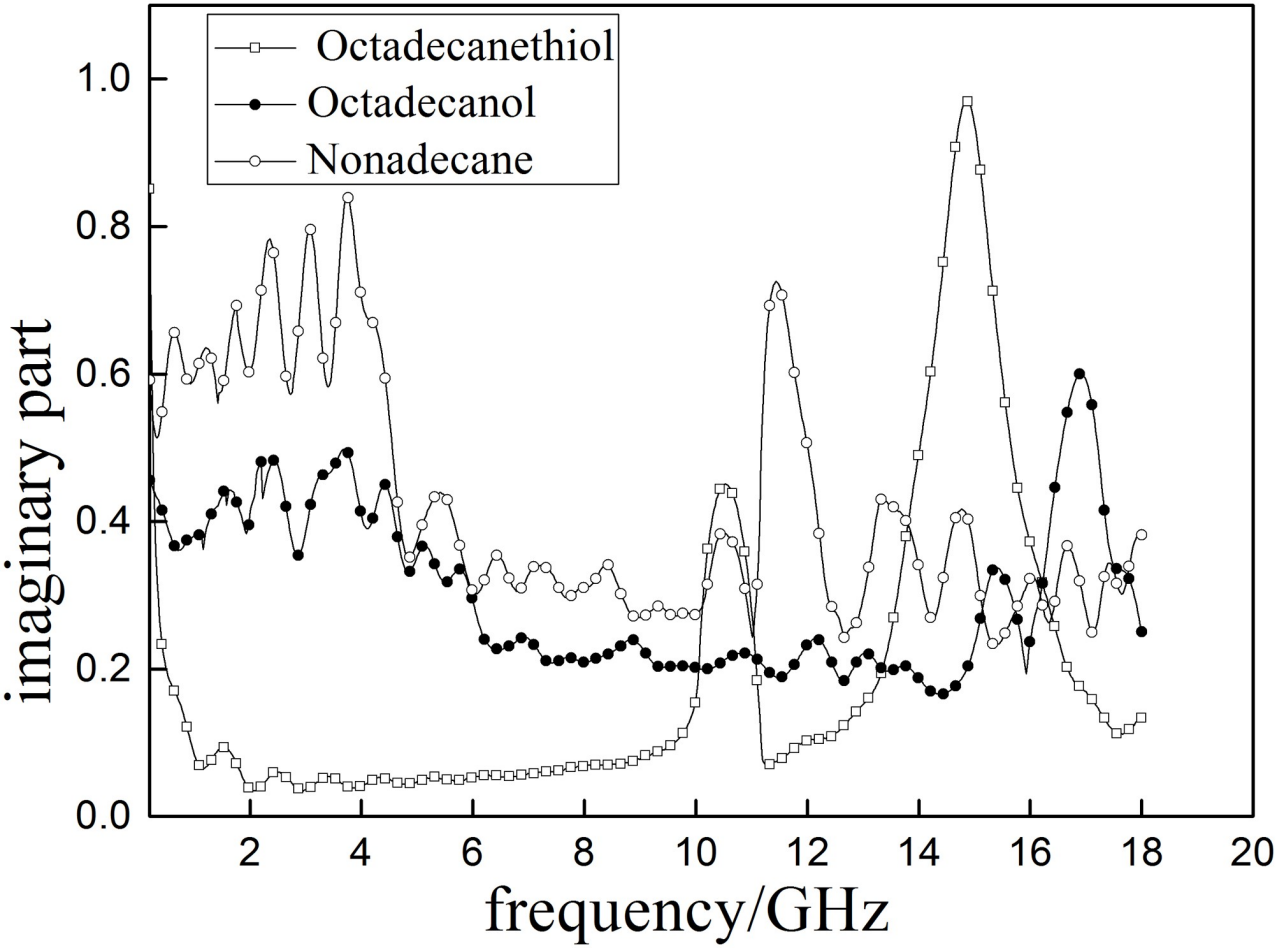
The imaginary part of the complex permeability (*ε*″) ofaliphatic model sulfur.

Dielectric properties of aliphatic model compounds are tested and shown in Figs 8 and 9. Results indicated that the *ε*′ in three model compounds are greatly different even with minimal changes of functional groups.

**Fig 10:**
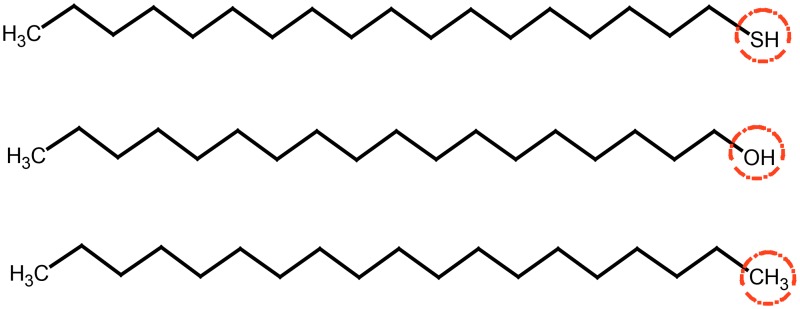
Molecular structures of aliphatic model compounds (from top to bottom are octadecyl mercaptan, stearyl alcohol and nonadecane).

*ε*′ of the sulfur-contain compound (octadecane thiol) are greater than 2.2 during the test range and shows two obvious peaks at 10.124 and 13.839 GHz. The *ε*′ of sulfur-free compounds (Octadecanol and nonadecane) are lower than 2.2 and also have peaks around 10GHz. In addition, Octadecanol and nonadecane also have peaks within2-4GHz. These differences between sulfur-contain and sulfur-free compounds are caused by their different structures as shown in Fig 10. The results indicate that the sulfur-contain functional groups can cause the absorption peak to shift to higher frequency.

### Aromatic model compounds

The molecular structures two of aromatic model compounds, dibenzothiophene and dibenzofuran, are shown in the Fig 10. Their molecular structures are very similar. The only difference dibenzofuran has an oxygen atom where dibenzothiophene has a sulfur atom.

**Fig 11:**
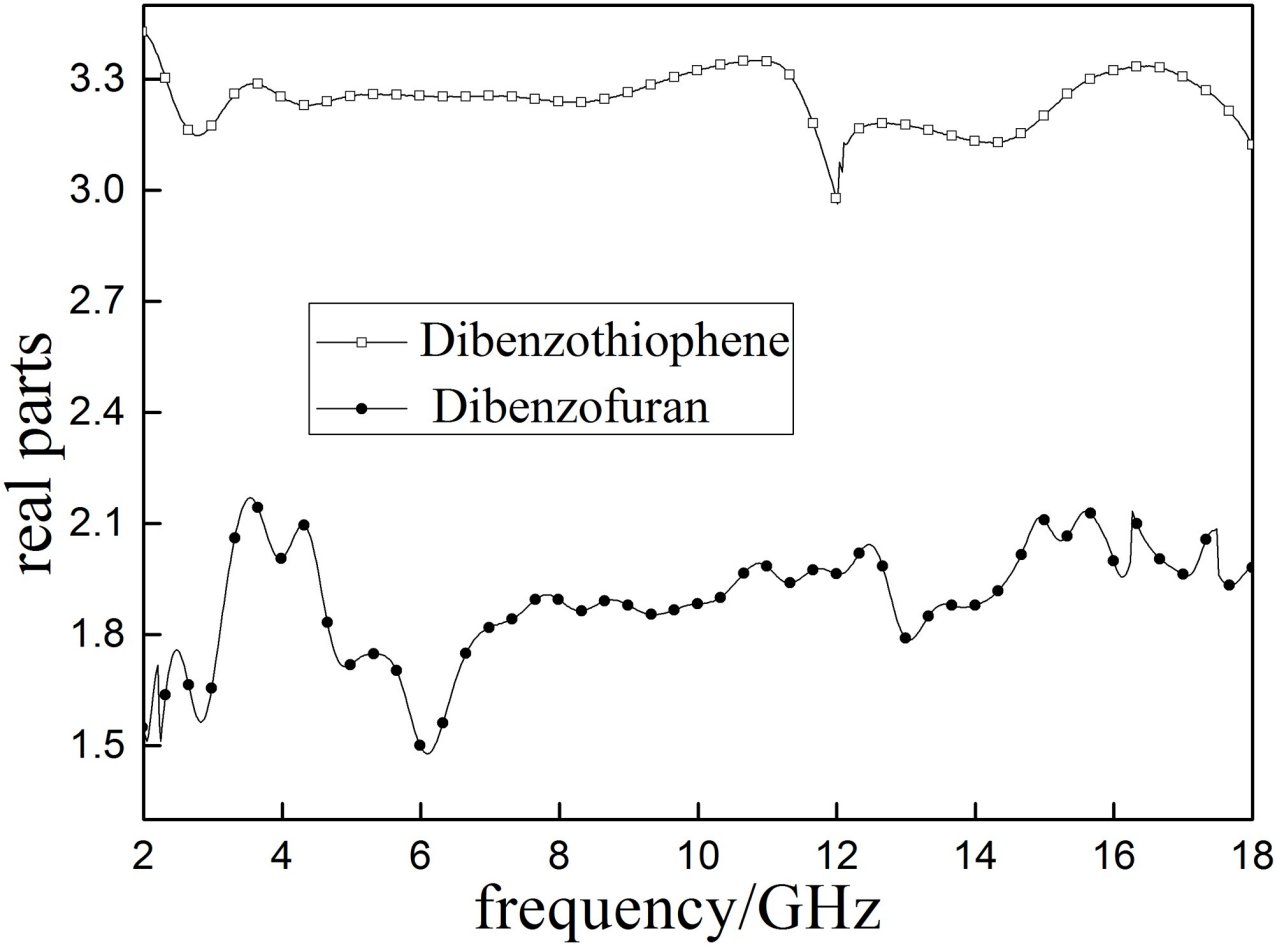
The real part (*ε*′)of the complex permeability of aromatic model sulfur compounds.

**Fig 12:**
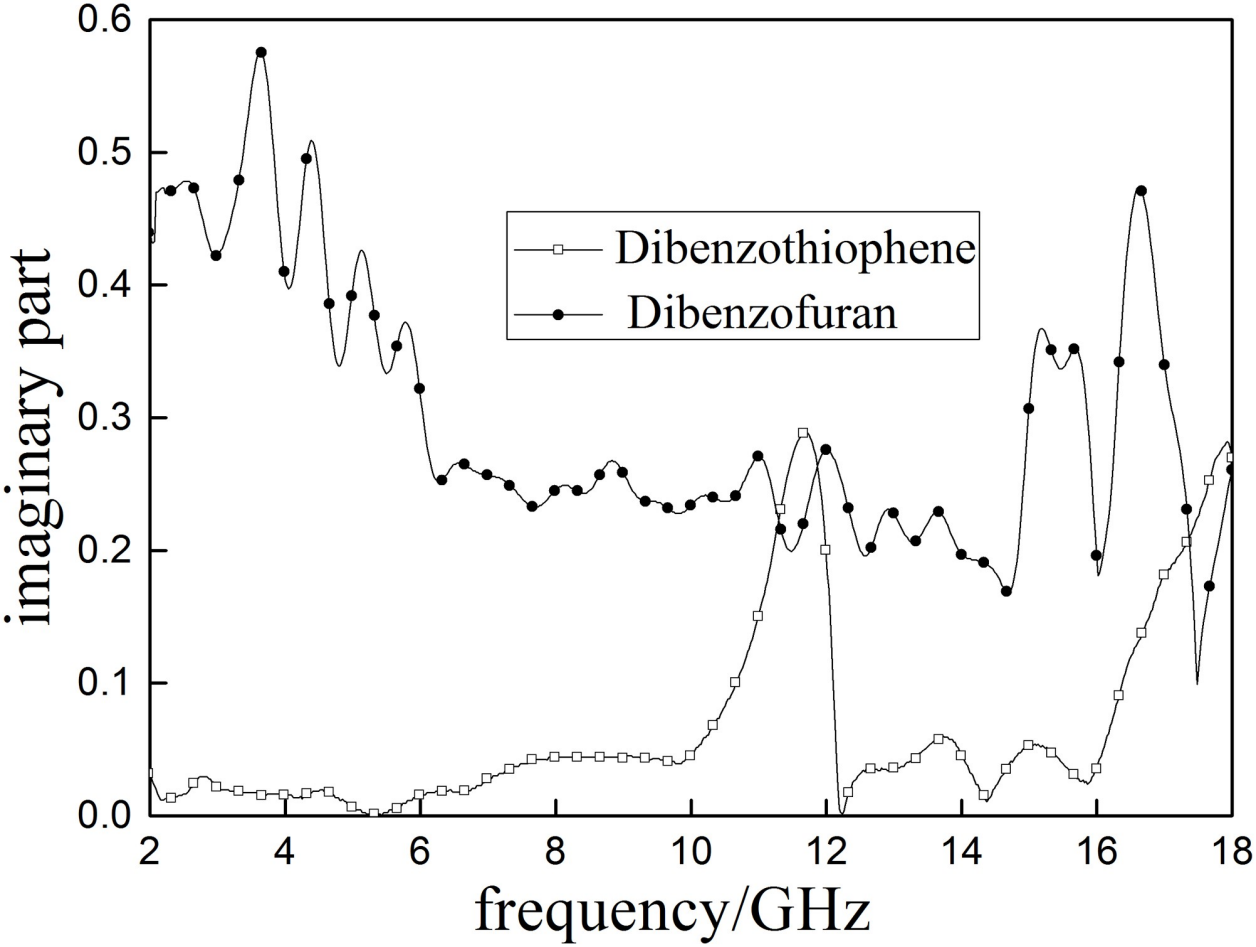
The imaginary part of the complex permeability (*ε*″) ofaromatic model sulfur compounds.

**Fig 13:**
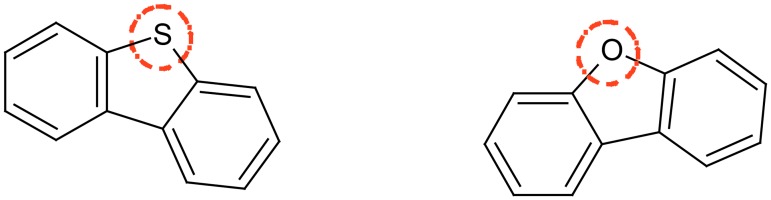
Molecular structures of aromatic model compounds (from left to right are dibenzothiophene and dibenzofuran).

Dielectric properties of aromatic model compounds are shown in Figs 11 and 12. Results indicatethat *ε*′ of dibenzothiophene (sulfur -contain) is significantly higher than that of dibenzofuran (oxygen-contain), suggesting that the specific sulfur bond in dibenzothiophene can increase the molecular polarity. The *ε*″ of dibenzothiophene is lower than dibenzofuran. Small changes in molecular structures (shown in Fig 13) can lead to big difference in their dielectric properties. This study also proved that involvement of sulfur in coal can greatly influence its polarity.

#### Dielectric properties of mixtures of sulfur model compounds and low-sulfur coal

**Fig 14:**
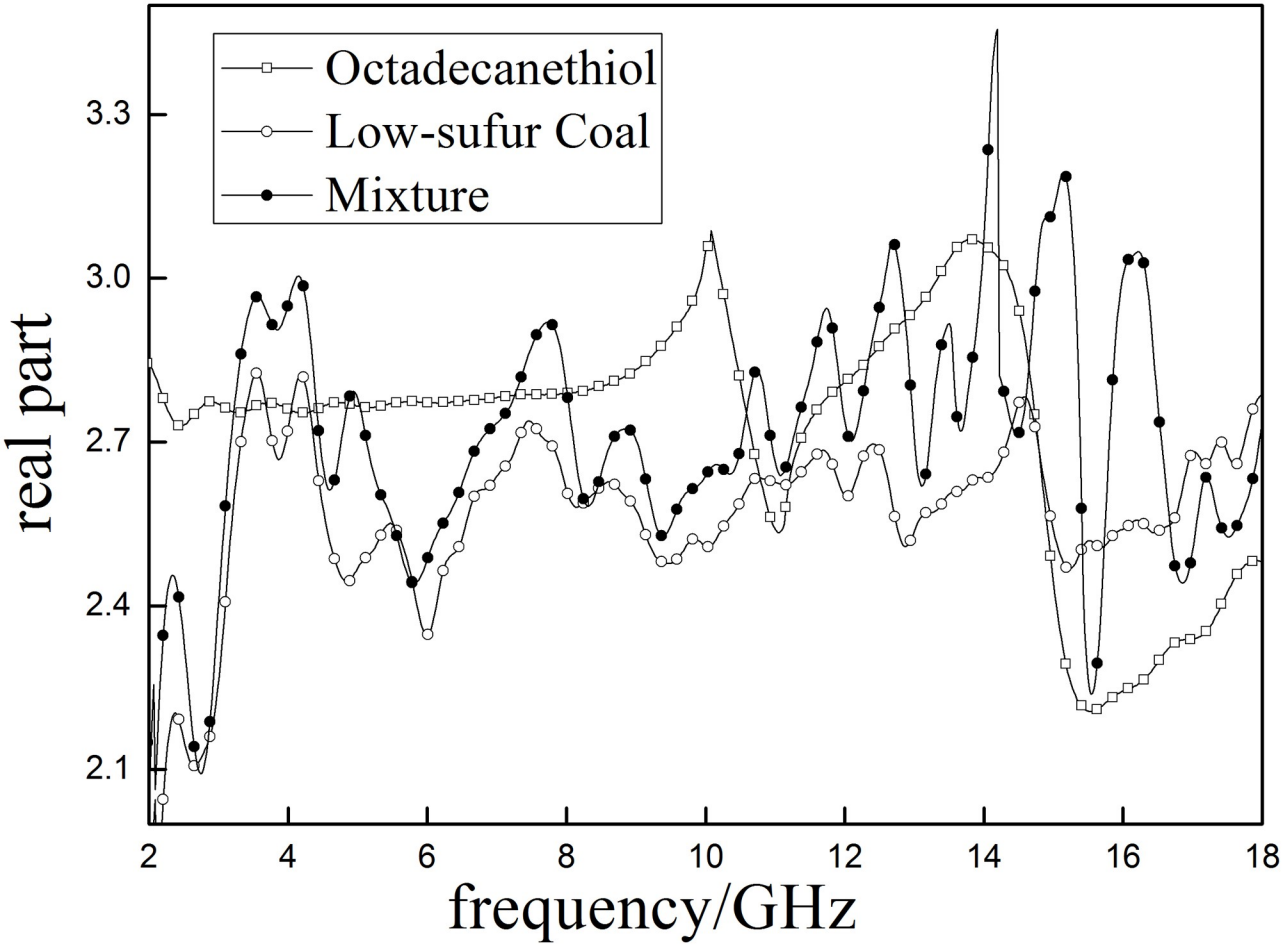
The real part (*ε*′)of low-sulfur coal, sulfur model compounds and the mixture.

**Fig 15:**
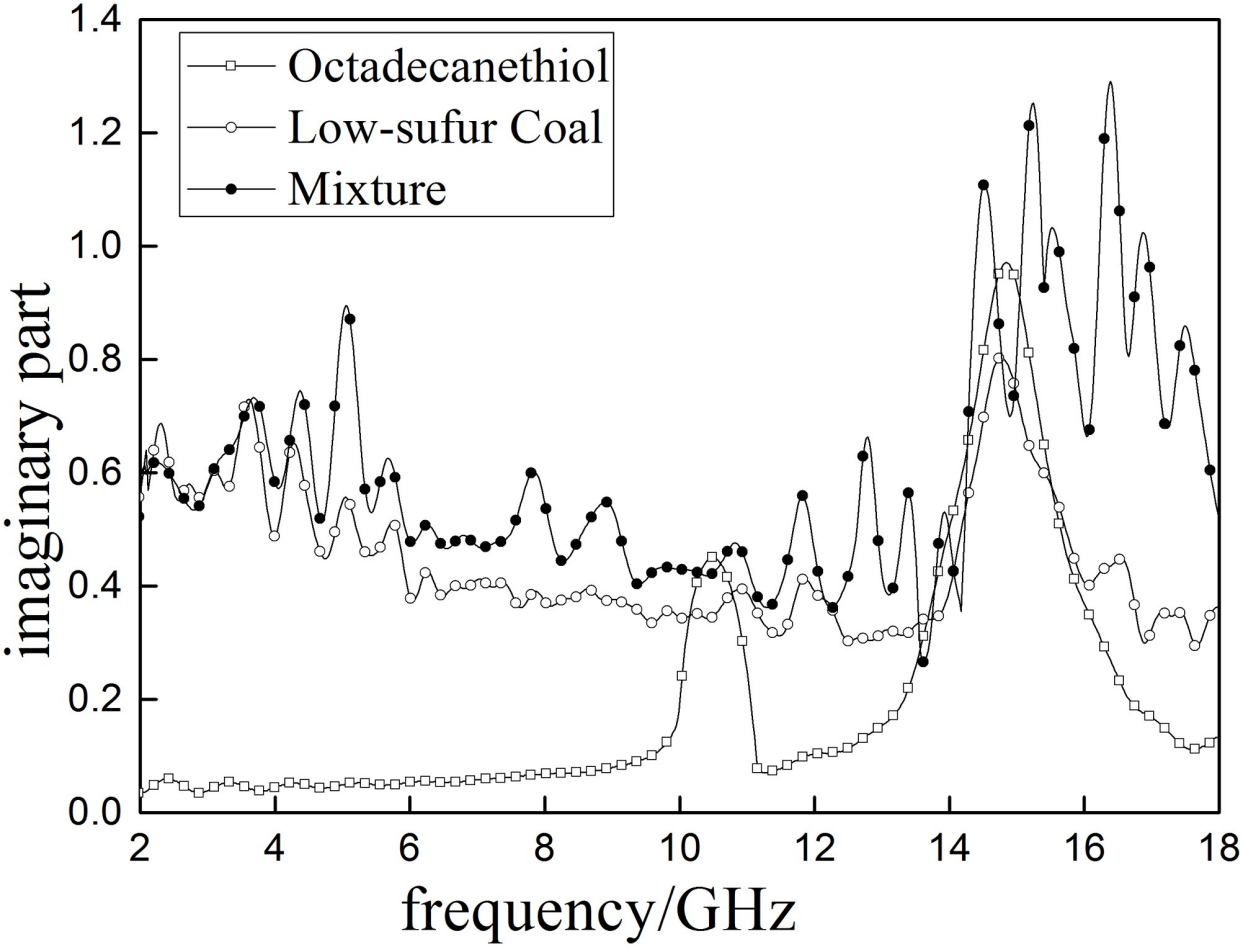
The imaginary part of the complex permeability (*ε*″) oflow-sulfur coal, sulfur model compounds and the mixture.

Dielectric properties of low-sulfur coal and low-sulfur coal mixed with selected sulfur model compounds are shown in Figs 14 and 15.

As shown in Figs 14 and 15, there are significant differences between low sulfur coal (Coal D), the selected sulfur model compounds (octadecanethiol) and their mixture. The *ε*′ curve of octadecanethiol has two obvious peaks (10.524GHz and 14.863GHz), however, the coal and mixture have more than two peaks. The *ε*′ of octadecanethioland the mixture are greater than that of Coal D, indicating that the selected sulfur model compound can increase the polarity of Coal D. In addition, the *ε*″ of mixture is higher than pure coal, suggesting that octadecanethiol can help increase the capability of microwave absorption of coal.

## Conclusion

1. The main types of organic sulfur in selected coal samples are mercaptan, thiophene, and sulfone. The content of thiopheneis higher than mercaptan and sulfone in Coal B and Coal C. However, the content of these three types of organic sulfur is similar in Coal A.
2. Coal A has the greatest polarization and greatest response to microwave irradiation at 2.581 GHz. The *ε*″ of Coal A decreases first and then increases with the increase of frequency. The highest peak value of *ε*″ is 0.462 at 15.619 GHzand tan *α* is 0.093 at 15.664 GHz. The *ε*′, *ε*″ and tan *α* of Coal B and Coal C appear several peaks in 2-18GHz test frequency.
3. The *ε*′ of five major sulfur-containing model compounds decrease when the frequency increases. The trend of *ε*′ of sulfur model compounds is: diphenyl sulfone > diphenyl sulfoxide > diphenyl sulfide > dibenzothiophene > octadecane thiol. The *ε*′ and *ε*″ of sulfur-containing compounds are higher than those of sulfur-free compounds with similar structure. Oxidation treatment can improve the sample’s response to microwave energy and enhance the heating efficiencyat certain frequency.
4. The selected sulfur model compound can increase the polarity of Coal D. Introduction of sulfur-containing model compounds can help improve the capability of microwave absorption of coal.

## Supporting information

S1 File Data for Fig 2. (XLSX)

S2 File Data for Figs 3, 4 and 5. (XLSX)

S3 File Data for Figs 6 and 7. (XLSX)

S4 File Data for Figs 8 and 9. (XLSX)

S5 File Data for Figs 11 and 12. (XLSX)

S6 File Data for Figs 14 and 15. (XLSX)
